# Ultrasound-Assessed Brachial Artery Flow-Mediated Dilation and Carotid Plaque Burden as Markers of Vascular Health in Relation to Weekly Physical Activity Duration in Older Adults

**DOI:** 10.3390/diagnostics16081229

**Published:** 2026-04-20

**Authors:** Michał Fułek, Tomasz Harych, Piotr Macek, Katarzyna Fułek, Krzysztof Kraik, Barbara Dziadkowiec-Macek, Jarosław Domaradzki, Małgorzata Poręba, Andrzej Wysocki, Paweł Gać, Rafał Poręba

**Affiliations:** 1 Department of Diabetology, Hypertension and Internal Diseases, Institute of Internal Diseases, Wroclaw Medical University, 50-556 Wroclaw, Poland; 2 Department of Recreation and Tourism, Wroclaw University of Health and Sport Sciences, 51-617 Wroclaw, Poland; tomasz.harych@awf.wroc.pl; 3 Department of Environmental Health, Occupational Medicine and Epidemiology, Wroclaw Medical University, 50-345 Wroclaw, Poland; piotr.macek@umw.edu.pl; 4 Department of Cardiology, Marciniak Lower Silesian Specialist Hospital, 54-049 Wroclaw, Poland; 5 Department of Otolaryngology, Wroclaw Medical University, 50-556 Wroclaw, Poland; katarzyna.fulek@umw.edu.pl; 6 Faculty of Medicine, Wroclaw Medical University, 50-345 Wroclaw, Poland; krzysztof.kraik@gmail.com; 7 Division of Pathophysiology, Department of Physiology and Pathophysiology, Wroclaw Medical University, 50-368 Wroclaw, Poland; dziadkowiecbarbara@gmail.com; 8 Department of Biological Principles of Physical Activity, Wroclaw University of Health and Sport Sciences, 51-612 Wroclaw, Poland; jaroslaw.domaradzki@awf.wroc.pl (J.D.); malgorzata.poreba@awf.wroc.pl (M.P.); rafal.poreba@awf.wroc.pl (R.P.); 9 Department of Radiology and Diagnostic Imaging, 4th Military Hospital, 50-981 Wroclaw, Poland; awysocki@4wsk.pl

**Keywords:** physical activity, flow-mediated dilation, carotid atherosclerosis, endothelial function, older adults, geriatrics

## Abstract

**Objectives**: This study assessed the association between physical activity and vascular endothelial function and carotid atherosclerotic plaques in older adults using ultrasound imaging. **Methods and Results**: A total of 60 older adults were divided into three groups based on weekly physical activity: low (<4 h/week), moderate (4–12 h/week), and high (>12 h/week). All participants underwent flow-mediated dilation (FMD) assessment and carotid ultrasound to determine plaque burden. Participants with moderate physical activity demonstrated greater FMD (12.3 ± 2.5%) than those with low (1.2 ± 1.8%) or high activity (7.1 ± 1.3%), and a lower carotid plaque burden. In adjusted logistic regression models, moderate activity remained independently associated with a lower likelihood of impaired FMD (OR 0.22; 95% CI 0.18–0.27) and lower carotid plaque burden (OR 0.16; 95% CI 0.11–0.19). **Conclusions**: Moderate physical activity was associated with more favorable vascular endothelial function and reduced carotid atherosclerosis in older adults, indicating an association with a more favorable vascular profile.

## 1. Introduction

### 1.1. Background on Aging and Cardiovascular Risk

Aging leads to a decline in vascular function and integrity [1], significantly contributing to cardiovascular diseases [2,3], the leading cause of global morbidity and mortality [4,5]. In elderly individuals, endothelial dysfunction, increased arterial stiffness, and a higher prevalence of atherosclerotic changes are commonly observed, significantly reducing healthy life expectancy [6]. These alterations result from complex interactions between genetic predispositions, chronic low-grade inflammation, oxidative stress, and cumulative exposure to cardiovascular risk factors over time. As a consequence, the aging vascular system becomes more susceptible to functional impairments and structural remodeling, promoting the onset and progression of atherosclerosis [7]. This strong interconnection between systemic inflammation, oxidative stress, and aging has led to the emergence of a dedicated field of research known as inflammaging, which aims to elucidate the molecular and cellular mechanisms underlying age-related vascular deterioration [8]. Understanding modifiable factors that can attenuate these changes is essential for improving vascular health and reducing CVD risk in older adults.

### 1.2. Endothelial Function and Atherosclerosis—Clinical Relevance

The endothelium plays a pivotal role in maintaining vascular homeostasis by regulating vascular tone, inflammation, hemostasis, and permeability [9]. Endothelial dysfunction is considered an early marker and key contributor to the development of atherosclerosis [10]. One of the most widely used non-invasive methods for assessing endothelial function is flow-mediated dilation (FMD) of the brachial artery, which reflects nitric oxide-dependent vasodilation in response to shear stress [11]. Impaired FMD has been independently associated with increased cardiovascular risk and adverse outcomes in various populations [12]. In parallel, the presence and extent of atherosclerotic plaques in the carotid arteries, as visualized by ultrasound, serve as reliable markers of systemic atherosclerosis and predictors of future cardiovascular events [13]. It also correlates with the severity of coronary artery disease, suggesting its potential role as a surrogate marker of coronary atherosclerotic burden, particularly in hypertensive patients [14]. Therefore, together FMD and carotid plaque burden provide complementary insights into vascular health and subclinical atherosclerotic disease progression [15].

### 1.3. Role of Physical Activity in Vascular Health

Regular physical activity is one of the most effective lifestyle interventions for the prevention and management of cardiovascular disease [16]. It exerts multiple beneficial effects on vascular health by improving endothelial function, reducing arterial stiffness, lowering blood pressure, enhancing lipid profiles, and mitigating systemic inflammation [17,18]. Exercise-induced shear stress stimulates endothelial nitric oxide production, thereby enhancing vasodilation and promoting anti-atherogenic effects [19,20]. Numerous studies have demonstrated that physically active individuals exhibit better flow-mediated dilation and reduced progression of carotid atherosclerosis compared to sedentary peers [21,22]. However, the relationship between the weekly physical activity duration and vascular outcomes remains complex. While moderate levels of exercise appear to confer optimal vascular benefits, some reports suggest that very high-intensity activity may not provide additional advantages and could even induce transient vascular stress [23]. This underscores the importance of identifying the most favorable level of physical activity for preserving vascular health, particularly in older adults.

### 1.4. Rationale for the Study

Despite extensive evidence supporting the cardiovascular benefits of physical activity, the optimal intensity of exercise for promoting vascular health in older adults remains a subject of ongoing debate [24]. Many existing studies have compared less active and more active individuals, while the relationship between different levels of weekly physical activity duration and vascular outcomes in older adults remains insufficiently characterized [23]. Furthermore, there is limited data specifically addressing how different levels of physical activity influence both functional (e.g., endothelial responsiveness assessed by FMD) and structural (e.g., carotid plaque burden) markers of vascular health in the aging population. Moreover, most available studies do not differentiate between time spent on physical activity and its physiological intensity, which may have distinct effects on vascular outcomes. Given the increasing proportion of elderly individuals worldwide and their heightened risk of atherosclerotic cardiovascular disease [25,26], there is a pressing need to better understand how varying degrees of physical activity impact the vasculature in this group.

### 1.5. Objectives

The primary objective of this study was to assess the association between weekly physical activity duration and vascular health in older adults, as measured by two complementary ultrasound-based markers: flow-mediated dilation (FMD) of the brachial artery and carotid atherosclerotic plaque burden. Specifically, we aimed to compare these parameters across three groups of older individuals categorized according to self-reported weekly physical activity duration: low, moderate, and high. By examining both functional and structural indicators of vascular status, this study sought to determine whether different levels of weekly physical activity duration were associated with differences in endothelial function and subclinical atherosclerosis in the aging population.

## 2. Materials and Methods

### 2.1. General Characteristics of the Performed Study

This cross-sectional observational study was conducted on a cohort of older adults recruited between 1 April 2023 and 30 November 2023 from outpatient cardiology settings in Wroclaw, Poland. The final study sample comprised 60 participants. Group size was determined using a sample size calculator. The selection conditions were as follows: population size 2.8 million, corresponding to the population from which participants were recruited for the study, that is, the population of the Lower Silesian Voivodeship in Poland; fraction size 0.19, reflecting the approximate proportion of older adults in the population of the Lower Silesian Voivodeship, maximum error 10% and confidence level 95%. Based on these assumptions, the required minimum sample size was 60 participants. The study protocol was reviewed and approved by the Bioethics Committee of Wroclaw Medical University (Approval No. KB-210/2023), in accordance with the principles outlined in the Declaration of Helsinki. The design and reporting of the study adhered to the Strengthening the Reporting of Observational Studies in Epidemiology (STROBE) guidelines [27], which provide a standardized framework for observational research. All participants provided written informed consent after receiving comprehensive information about the study’s objectives, procedures, and potential implications.

### 2.2. Eligibility Criteria

Eligibility criteria included age > 55 years and the ability to provide written informed consent and comply with study procedures. A total of 97 individuals were initially screened for participation during routine visits to the outpatient cardiology clinic. We excluded participants with the following conditions: prior myocardial infarction (*n* = 8), prior stroke (*n* = 6), diagnosed heart failure (*n* = 8), history of cardiac or vascular surgery (*n* = 7), major surgery within the preceding six months (*n* = 4), severe valvular heart disease (*n* = 6), history of malignancy (*n* = 16), autoimmune or chronic inflammatory diseases (*n* = 5), use of immunosuppressive medications (*n* = 4), and nitrate therapy (*n* = 5). Several individuals met more than one exclusion criterion. After applying all predefined exclusion criteria, 60 participants remained eligible and were included in the final analysis. The aim was to obtain a relatively homogeneous cohort of older adults without overt clinical cardiovascular disease, in whom subclinical vascular alterations in relation to physical activity levels could be more reliably assessed.

### 2.3. Physical Activity Assessment

Participants were assigned to one of three groups based on their self-reported weekly physical activity duration, assessed through a study-specific structured interview conducted by trained personnel. The classification was as follows: Group A—low physical activity (<4 h per week), Group B—moderate physical activity (4–12 h per week), and Group C—high physical activity (>12 h per week). The reported activity included both structured forms of exercise (e.g., walking, cycling, swimming, gym-based training) and regular leisure-time physical activity. Participants were asked to estimate the average number of hours spent on physical activity per week over the preceding three months. This approach allowed for a practical stratification of the cohort while reflecting real-life variability in exercise behavior among older adults. However, this method does not capture important dimensions such as exercise intensity, modality, or training load, and therefore the classification reflects only weekly time spent on physical activity rather than standardized activity categories. As a result, the “high physical activity” group (>12 h/week) may still encompass heterogeneous patterns of leisure-time activity with varying physiological impact. Physical activity was assessed solely based on self-reported weekly hours during the preceding three months.

### 2.4. Flow-Mediated Dilation (FMD) Measurement

Endothelial function was evaluated non-invasively using the flow-mediated dilation (FMD) technique, following established guidelines [12,28]. The measurement was performed in a quiet, temperature-controlled room, with the participant resting in a supine position for at least 10 min prior to the examination. A high-resolution ultrasound device equipped with a linear transducer (7–12 MHz) was used to visualize the brachial artery in the distal third of the dominant arm.

Baseline brachial artery diameter (BAD) was measured at rest. Subsequently, a blood pressure cuff placed on the forearm was inflated to 50 mmHg above the systolic blood pressure and maintained for 5 min to induce ischemia. Following rapid cuff deflation, reactive hyperemia occurred, and the maximum post-occlusion artery diameter was recorded within 60–90 s. The FMD value was expressed as a percentage increase from the baseline diameter using the following formula:

FMD (%) = [(maximum diameter − baseline diameter)/baseline diameter] × 100.

All FMD measurements were performed by the same experienced sonographer (R.P.). The estimated reproducibility of FMD was approximately 90%. Repeatability was assessed by comparing the differences between two measurements obtained in each patient at 15 min intervals, taking into account the limits of agreement defined as the arithmetic mean ± 2 standard deviations. Approximately 90% of the observed differences in FMD values were within these limits of agreement.

### 2.5. Carotid Ultrasound and Plaque Burden Evaluation

The Carotid ultrasound examinations were performed using the same high-resolution ultrasound system and linear transducer employed for FMD assessment. The evaluation included bilateral imaging of the common carotid artery (CCA), carotid bulb, and internal carotid artery (ICA). Patients were examined in the supine position with slight hyperextension and contralateral rotation of the neck to optimize vessel visualization.

Atherosclerotic plaques were defined as focal structures encroaching into the arterial lumen by at least 0.5 mm, or 50% of the surrounding intima-media thickness (IMT), or having a thickness >1.5 mm as measured from the intima–lumen interface to the media-adventitia interface. The carotid plaque burden was quantified by counting the number of arterial segments (CCA, bulb, ICA) in which plaques were detected on either side.

Plaque assessment was conducted by an experienced operator (R.P.) blinded to the participants’ physical activity status. Carotid plaque burden was assessed using a semi-quantitative segment-based approach, defined as the number of arterial segments with detectable plaque. This method allowed estimation of total atherosclerotic load in the carotid circulation; however, it did not account for plaque size, volume, or detailed morphological characteristics.

### 2.6. Clinical and Biochemical Data

Basic clinical parameters were recorded for all participants, including age, sex, height, body weight, and body mass index (BMI), calculated as weight in kilograms divided by height in meters squared. Blood pressure was measured using a calibrated sphygmomanometer after at least 5 min of rest in the seated position. Two consecutive readings were taken, and the average value was used in the analysis.

Venous blood samples were collected in the morning after an overnight fast. Biochemical analyses included fasting plasma glucose, total cholesterol, and triglyceride concentrations, measured using standard enzymatic methods at a certified laboratory. Additional clinical data such as the presence of arterial hypertension, type 2 diabetes mellitus, and current smoking status were obtained through medical history and patient interview. Additional clinical information was collected to better characterize potential confounding factors related to endothelial function and atherosclerosis. These included the use of antihypertensive medications (ACE inhibitors, β-blockers, diuretics, calcium channel blockers, angiotensin receptor blockers), antidiabetic therapies (oral hypoglycemic agents, insulin, or combination therapy), and lipid-lowering medications (statins or fibrates). Detailed smoking data were also obtained, including current smoking status, the number of cigarettes smoked per 24 h, years of smoking, and calculated “smoking years.” All these variables were incorporated into subsequent analyses as potential cardiovascular risk modifiers.

These parameters were used to characterize the study population and to explore potential correlations with vascular function and atherosclerotic plaque burden.

### 2.7. Statistical Analysis

Statistical analyses were conducted using the Dell Statistica 13.1 software package (Dell Inc., Round Rock, TX, USA). Descriptive statistics for continuous variables included means and standard deviations (SD). The distribution of each variable was assessed using the Shapiro–Wilk test with the Lilliefors correction, and homogeneity of variances was evaluated using Levene’s and Brown–Forsythe tests. Depending on the distribution characteristics, comparisons across the three physical activity groups were performed using one-way ANOVA for normally distributed variables with homogeneous variances or the Kruskal–Wallis test for non-parametric data. When global tests were statistically significant, post hoc pairwise comparisons were carried out using the Newman–Keuls test. Categorical variables were summarized as counts and percentages.

Relationships between continuous variables were assessed using Pearson correlation coefficients. Variables presented in Table 1 (including body mass index, systolic and diastolic blood pressure, fasting glucose, lipid profile, type of cardiometabolic treatment, and detailed smoking exposure) were evaluated as potential covariates. Multivariable logistic regression models were constructed to determine whether physical activity level remained independently associated with endothelial function (FMD below vs. above the cohort mean) and carotid plaque burden (plaque score above vs. at or below the cohort mean) after adjustment for these confounders. A *p*-value < 0.05 was considered statistically significant throughout the analysis.

## 3. Results

### 3.1. General Characteristics of the Study Population

The study involved 60 adults with a mean age of 63.95 ± 8.96 years. The mean body height was 168.55 ± 10.01 cm, and the average body weight was 78.58 ± 14.54 kg, resulting in a mean body mass index (BMI) of 27.61 ± 4.34 kg/m^2^. Women constituted 56.7% of the study group, while men accounted for 43.3%.

Based on self-reported weekly physical activity, participants were classified into three groups: Group A—low activity (<4 h/week, 33.3%), Group B—moderate activity (4–12 h/week, 36.7%), and Group C—high activity (>12 h/week, 30.0%).

Arterial hypertension was present in 40.0% of participants, with average systolic and diastolic blood pressures of 128.60 ± 14.45 mmHg and 78.69 ± 8.82 mmHg, respectively. Type 2 diabetes mellitus was diagnosed in 11.7% of subjects. The mean fasting glucose level was 103.67 ± 16.57 mg/dL. Mean total cholesterol and triglyceride concentrations were 188.38 ± 43.22 mg/dL and 114.52 ± 42.23 mg/dL, respectively. Additionally, 25.0% of participants reported current smoking status.

These clinical and metabolic data are summarized in Table 1.

Table 1 includes detailed clinical information, including antihypertensive, antidiabetic, and lipid-lowering therapies, as well as quantitative smoking data (cigarettes/24 h, years of smoking, smoking years), collected to characterize cardiometabolic risk and potential confounding factors affecting vascular outcomes.

### 3.2. Vascular Parameters in the Whole Cohort

In the total study population, the mean baseline brachial artery diameter (BAD) was 4.23 ± 0.80 mm, while the maximum post-occlusion diameter reached 4.51 ± 0.79 mm. The average absolute change in artery diameter following reactive hyperemia was 0.28 ± 0.20 mm, corresponding to a mean flow-mediated dilation (FMD) of 7.01 ± 5.08%. The mean time to FMD peak was 68.80 ± 64.15 s.

The assessment of structural vascular changes revealed a mean carotid plaque score of 1.95 ± 1.52, representing the average number of arterial segments with atherosclerotic plaques across the common carotid artery, carotid bulb, and internal carotid artery.

These findings provide a general overview of vascular function and subclinical atherosclerosis in the study cohort and are summarized in Table 2.

### 3.3. Comparison of Vascular Markers Between Physical Activity Groups

Significant differences were observed across physical activity groups in both endothelial function and carotid atherosclerotic burden (Table 3).

**Figure 1:**
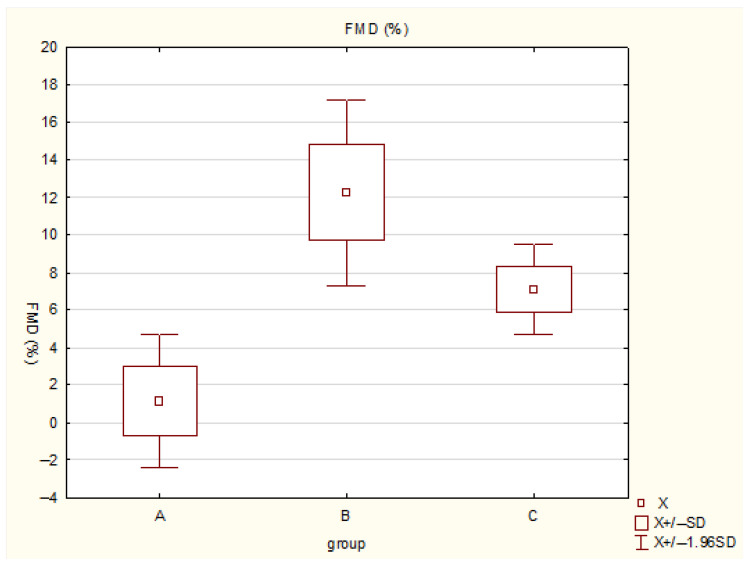
Comparison of flow-mediated dilation (FMD) values across physical activity groups. Group A: low physical activity (<4 h/week); Group B: moderate physical activity (4–12 h/week); Group C: high physical activity (>12 h/week). The plot displays mean values with standard deviations (±SD) and 95% confidence intervals (±1.96 SD). The highest FMD was observed in the moderate activity group (B), indicating superior endothelial function.

Participants in Group B (moderate activity) demonstrated the most favorable vascular profile, with a markedly higher mean FMD (12.27 ± 2.53%) compared with both Group A (1.15 ± 1.84%, *p* < 0.05) and Group C (7.10 ± 1.25%, *p* < 0.05). The lowest FMD values were observed in Group A (Figure 1).

**Figure 2:**
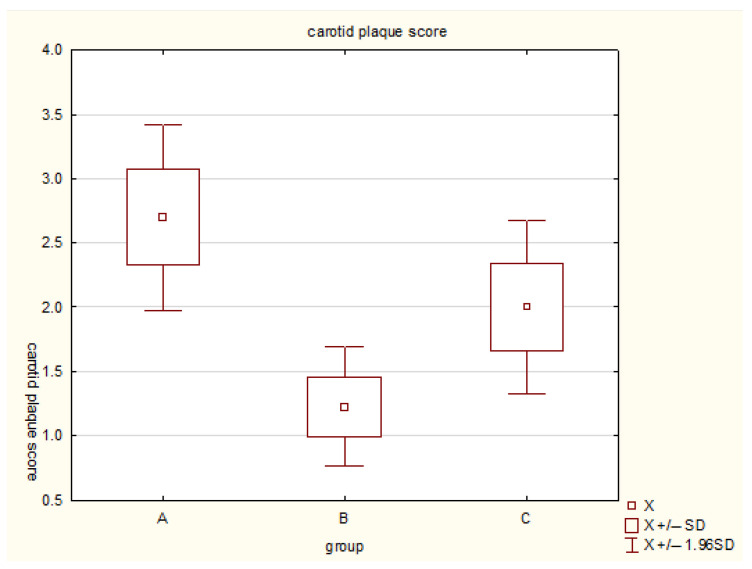
Comparison of carotid plaque score across physical activity groups. Group A: low physical activity (<4 h/week); Group B: moderate physical activity (4–12 h/week); Group C: high physical activity (>12 h/week). The plot shows mean values with standard deviations (±SD) and 95% confidence intervals (±1.96 SD). The lowest plaque burden was observed in the moderate activity group (B), indicating an association with lower carotid plaque burden.

Similarly, carotid plaque burden was significantly lower in Group B (1.23 ± 1.11) than in Group A (2.70 ± 1.66, *p* < 0.05) and Group C (2.01 ± 1.46, *p* < 0.05). The highest plaque scores were recorded in Group A (Figure 2).

Overall, moderate levels of physical activity were associated with the most favorable vascular function and the lowest degree of subclinical carotid atherosclerosis.

### 3.4. Correlation Analysis Between Clinical Variables and Vascular Parameters

Correlation analysis revealed several statistically significant associations between clinical parameters and vascular measurements (Table 4). Flow-mediated dilation (FMD) demonstrated significant inverse correlations with age (r = −0.35), BMI (r = −0.25), systolic blood pressure (r = −0.42), diastolic blood pressure (r = −0.48), fasting glucose (r = −0.41), total cholesterol (r = −0.31), and triglyceride levels (r = −0.28). These findings suggest that impaired endothelial function is associated with advancing age, increased adiposity, elevated blood pressure, and adverse metabolic profiles.

Conversely, carotid plaque score correlated positively with several cardiovascular risk factors, including BMI (r = 0.34), systolic blood pressure (r = 0.38), diastolic blood pressure (r = 0.29), total cholesterol (r = 0.48), and triglycerides (r = 0.37). These relationships reflect the cumulative impact of metabolic and hemodynamic stressors on structural vascular changes in the carotid arteries.

Collectively, these correlations support the role of traditional cardiovascular risk factors in modulating both functional and structural indicators of vascular health in older adults.

### 3.5. Multivariable Logistic Regression Analysis

Predictors of Impaired Endothelial Function are presented in Table 5. In the multivariable model evaluating the probability of having an FMD value below the cohort mean, moderate physical activity remained a significant independent predictor of better endothelial function (regression coefficient −2.61; OR 0.22; 95% CI 0.18–0.27; *p* = 0.03). Higher systolic blood pressure also independently predicted reduced endothelial function (Rc 0.09; OR 1.11; 95% CI 1.05–1.16; *p* = 0.02), as did current smoking (Rc 2.68; OR 4.48; 95% CI 3.18–5.81; *p* = 0.03). These results indicate that the beneficial association between moderate physical activity and endothelial function persists even after adjustment for major cardiovascular risk factors and smoking exposure.

Predictors of Higher Carotid Plaque Burden are presented in Table 6. In the multivariable model assessing the probability of having a carotid plaque score above the cohort mean, moderate physical activity remained independently associated with a lower likelihood of advanced carotid atherosclerosis (Rc −2.39; OR 0.16; 95% CI 0.11–0.19; *p* = 0.04). Independent predictors of greater plaque burden included:—higher BMI (Rc 4.42; OR 1.23; 95% CI 1.11–1.31; *p* = 0.04),—higher total cholesterol (Rc 0.41; OR 1.05; 95% CI 1.03–1.08; *p* = 0.01),—current smoking (Rc 4.01; OR 4.61; 95% CI 3.97–5.40; *p* = 0.02). These findings indicate that moderate physical activity was independently associated with a lower likelihood of advanced carotid atherosclerosis.

## 4. Discussion

### 4.1. Summary of Key Findings

This study demonstrated a significant association between the weekly physical activity duration and both functional and structural markers of vascular health in older adults. Individuals engaging in moderate levels of weekly physical activity (4–12 h/week) exhibited the most favorable vascular profile, characterized by the highest flow-mediated dilation (FMD) values and the lowest carotid plaque burden. In contrast, participants with low physical activity levels (<4 h/week) showed markedly impaired endothelial function and a higher extent of carotid atherosclerosis. In this context, the mean FMD observed in Group A may be interpreted as reflecting a markedly impaired endothelial profile, whereas the value observed in Group B indicates a substantially more favorable vascular phenotype. Interestingly, individuals in the high-activity group (>12 h/week) did not show further vascular improvement compared with the moderate-activity group. However, this finding should be interpreted cautiously, as the >12 h/week category may have included heterogeneous activity types with differing physiological intensity and training load.

Moderate levels of physical activity were associated with a more favorable vascular profile in older adults, unlike insufficient or excessive activity levels. Given the non-standardized and self-reported nature of physical activity assessment, these findings should be interpreted as preliminary and hypothesis-generating.

The multivariable models, which incorporated detailed cardiovascular risk factors, pharmacotherapy, and smoking exposure (Table 1), confirmed that moderate physical activity remained a strong independent predictor of both better endothelial function and lower carotid plaque burden. Importantly, these associations persisted even after adjustment for systolic blood pressure, BMI, lipid profile, glucose levels, and smoking—variables that showed significant correlations with vascular measures in Table 4.

### 4.2. Clinical Implications

The results of this study offer important insights for cardiovascular prevention strategies in the aging population. While regular physical activity is widely recommended to maintain vascular health [29], our findings suggest that the intensity and volume of such activity are critical factors. Specifically, moderate weekly physical activity was associated with better endothelial function and lower atherosclerotic burden, potentially translating into a reduced risk of cardiovascular events.

These observations are consistent with a growing body of evidence suggesting that the relationship between physical activity and cardiovascular health may be non-linear, with some studies indicating a possible J-shaped pattern [30], particularly in older adults. However, our study was not designed to formally assess the shape of this association. In line with this concept, community-based programmes that promote regular activity may shift older adults toward a more favourable ‘dose’ of movement: in Poland, elderly women attending a University of the Third Age exhibited the most favourable functional biological health markers compared with less-active peers, underscoring the preventive potential of senior programmes that emphasise physical activity [31].

Numerous large-scale meta-analyses have demonstrated that even low volumes of physical activity are associated with a substantially reduced risk of all-cause mortality, though the magnitude of benefit plateaus at higher levels [30]. A similar L-shaped relationship has been reported for stroke risk in women, with diminishing returns at higher activity intensities, and a U-shaped pattern observed specifically for haemorrhagic stroke subtypes [32]. Moreover, meta-analyses of sedentary behaviour have revealed non-linear associations with cardiovascular mortality, where risk increases significantly beyond a daily threshold of 6–8 h of sitting time, independent of total physical activity [33]. In the context of arrhythmia risk, a J-shaped relationship has been observed between physical activity volume and atrial fibrillation, with moderate activity reducing risk, while higher volumes may negate this benefit [34].

Therefore, incorporating personalized physical activity recommendations into routine geriatric and preventive care may enhance cardiovascular outcomes, particularly when aligned with each patient’s functional capacity, comorbidities, and lifestyle preferences.

### 4.3. Pathophysiological Considerations

The observed benefits of moderate physical activity on vascular health in older adults are likely mediated by several interrelated physiological mechanisms. One of the key factors is shear stress-induced stimulation of endothelial nitric oxide (NO) production, which promotes vasodilation, inhibits platelet aggregation, and reduces inflammation within the vascular wall [35,36]. Regular, moderate-intensity exercise enhances this protective endothelial response, improving vascular tone and functional integrity.

In contrast, both sedentary behavior and excessive physical exertion may disrupt this balance. Physical inactivity contributes to oxidative stress, low-grade inflammation, and metabolic dysregulation, all of which impair endothelial function and accelerate atherogenesis. On the other hand, prolonged or intense exercise can transiently increase catecholamine levels, oxidative load, and arterial wall stress, which may paradoxically blunt NO bioavailability or promote vascular remodeling in susceptible individuals [37].

The non-linear relationship observed in this study supports the hypothesis that vascular benefits of exercise may plateau or even reverse beyond a certain weekly physical activity duration threshold, especially in older adults with underlying cardiovascular vulnerability. This highlights the need for an individualized and physiologically appropriate approach to physical activity prescription in this population.

### 4.4. Study Limitations and Directions for Future Research

Several limitations of this study should be acknowledged. First, the relatively small sample size may limit the generalizability of the findings and reduce the statistical power for detecting subtle differences between groups. Second, physical activity levels were assessed using self-reported data, which may be subject to recall bias, particularly given the 3-month recall period, and misclassification. Objective measurements, such as accelerometry, would provide more precise estimates of activity intensity and duration.

Third, the cross-sectional design of the study precludes causal inference. While associations between physical activity and vascular parameters were identified, longitudinal data are needed to confirm the directionality of these relationships and to assess the long-term effects of varying exercise intensities on vascular health. Additionally, we did not include direct biomarkers of inflammation or oxidative stress, which could further elucidate the underlying mechanisms linking physical activity with endothelial function and atherosclerosis. The absence of HbA1c measurements for participants with type 2 diabetes is an additional limitation, as it prevented a more detailed assessment of glycemic control and its potential impact on endothelial function or atherosclerosis. Residual confounding cannot be excluded, as unmeasured factors such as diet, fitness level, and socioeconomic status may also have influenced the observed associations.

In addition, FMD is an operator-dependent ultrasound parameter, and although repeatability in our study was estimated at approximately 90%, measurement-related variability cannot be fully excluded. Moreover, carotid plaque burden was assessed using a semi-quantitative segment-based score, which does not capture plaque size, volume, or detailed plaque morphology. Also, participants were recruited from outpatient cardiology settings, which may limit the representativeness of the study group for the general older adult population and introduce referral or selection bias.

Finally, an important methodological consideration concerns the lack of standardization of physical activity assessment. The grouping was based solely on self-reported weekly duration of activity, without the use of a validated physical activity questionnaire and without data on exercise intensity, modality, or training load. Device-based monitoring (e.g., accelerometry) was not used. As such, the “high physical activity” category may still represent heterogeneous leisure-time activities rather than a physiologically homogeneous high-intensity group. This heterogeneity reduces the generalizability of our findings and underscores the need for future studies employing validated questionnaires with intensity parameters or device-based objective monitoring.

Future research should aim to replicate these findings in larger, more diverse populations, preferably through prospective cohort studies or randomized controlled trials. Moreover, exploring sex-specific responses, the role of comorbidities, and individualized exercise prescriptions may enhance our understanding of how to optimize physical activity for vascular protection in older adults.

## 5. Conclusions

This study showed that moderate levels of weekly physical activity were associated with better endothelial function and lower carotid plaque burden in older adults. Participants engaging in 4–12 h of weekly physical activity demonstrated significantly better endothelial function and a lower burden of carotid atherosclerosis compared to those with low or high activity levels. Multivariable regression analyses indicated that moderate physical activity was independently associated with better endothelial function and lower carotid plaque burden in older adults, even after accounting for major cardiovascular risk factors and smoking exposure. These results are consistent with the statement that moderate physical activity is associated with the most favorable vascular profile, although larger prospective studies are needed to confirm this observation.

The results emphasize the importance of personalized, evidence-based physical activity recommendations in clinical practice. Rather than promoting the highest possible levels of exercise, clinicians should consider advising older patients to maintain a moderate and sustainable activity regimen to achieve the greatest cardiovascular benefits.

## Figures and Tables

**Table 1:** Clinical characteristics of the study cohort (*n* = 60).

	Whole Study Group (*n* = 60)
age (years)	63.95 ± 8.96
height (cm)	168.55 ± 10.01
body mass (kg)	78.58 ± 14.54
BMI (kg/m^2^)	27.61 ± 4.34
gender (%) men women	43.3 56.7
physical activity (%) <4 h/week 4–12 h/week >12 h/week	33.3 36.7 30.0
arterial hypertension (%)	40.0
systolic blood pressure (mmHg)	128.60 ± 14.45
diastolic blood pressure (mmHg)	78.69 ± 8.82
hypotensive drugs (%) ACE inhibitors β-blockers diuretics calcium channel blockers angiotensin receptor blockers	21.7 18.3 11.7 11.7 3.3
type 2 of diabetes (%)	11.7
fasting glucose (mg/dL)	103.67 ± 16.57
hypoglycemic therapy (%) oral hypoglycemic drugs insulin oral hypoglycemic drugs and insulin	5.0 1.7 5.0
total cholesterol (mg/dL)	188.38 ± 43.22
triglycerydes (mg/dL)	114.52 ± 42.23
hypolipedemic drugs (%) statins fibrates	31.7 18.3
smoking (%)	25.0
number of cigarettes/24 h years of smoking smoking years	24.67 ± 11.72 19.60 ± 9.12 473.01 ± 368.75

**Table 2:** Brachial artery flow-mediated dilation (FMD) and carotid plaque score in the study cohort (*n* = 60).

	Whole Study Group (*n* = 60)
native BAD (mm)	4.23 ± 0.80
maximum BAD after occlusion release (mm)	4.51 ± 0.79
FMD peak time SD (s)	68.80 ± 64.15
FMD peak time median (s)	60.0
FMD peak time interquartile range (s)	80.0
change in BAD (mm)	0.28 ± 0.20
FMD (%)	7.01 ± 5.08
carotid plaque score	1.95 ± 1.52

BAD—brachial artery diameter, FMD—flow-mediated dilation. Continuous variables are presented as mean ± standard deviation. Variables include: native brachial artery diameter (BAD), peak BAD after occlusion release, time to FMD peak, absolute change in BAD, relative FMD (%), and carotid plaque score (number of carotid segments with atherosclerotic plaque across both sides).

**Table 3:** Flow-mediated dilation (FMD) and carotid plaque score across study groups A–C.

	FMD (%)	Carotid Plaque Score
group A	1.15 ± 1.84	2.70 ± 1.66
group B	12.27 ± 2.53	1.23 ± 1.11
group C	7.10 ± 1.25	2.01 ± 1.46
*p*	A vs. B: <0.05 A vs. C: <0.05 B vs. C: <0.05	A vs. B: <0.05 B vs. C: <0.05

FMD—flow-mediated dilation.

**Table 4:** Correlations between clinical parameters and vascular measures (FMD and carotid plaque score).

	Age (Years)	BMI (kg/m^2^)	Systolic BP (mmHg)	Diastolic BP (mmHg)	Fasting Glucose (mg/dL)	Total Cholesterol (mg/dL)	Triglicerydes (mg/dL)
FMD (%)	**−0.35** ***p* < 0.045**	−0.25 *p* < 0.082	**−0.42** ***p* < 0.007**	**−0.48** ***p* < 0.002**	**−0.41** ***p* < 0.010**	−0.31 *p* < 0.064	−0.28 *p* < 0.074
carotid plaque score	0.11 *p* < 0.385	0.34 *p* < 0.052	**0.38** ***p* < 0.021**	0.29 *p* < 0.071	0.21 *p* < 0.119	**0.48** ***p* < 0.002**	**0.37** ***p* < 0.030**

Statistically significant correlations are bolded; BMI—body mass index, BP—blood pressure, FMD—flow-mediated dilation.

**Table 5:** Results of estimation for the final model for probability of “FMD < mean value” obtained in logistic regression analysis.

		Model for: Probability of “FMD < Mean Value”			
		Intercept	Physical Activity *	Systolic Blood Pressure (mmHg)	Smoking **
Regression coefficient		−5.89	−2.61	0.09	2.68
SEM of Rc		1.89	1.22	0.03	1.26
*p*-value		0.03	0.03	0.02	0.03
Odds ratio (for unit change)		0.02	0.22	1.11	4.48
Confidence interval					
	−95%	0.01	0.18	1.05	3.18
	+95%	0.05	0.27	1.16	5.81

* ordinal variable, where: 0—low or high activity, 1—moderate activity, ** ordinal variable, where: 0—no, 1—yes, FMD—flow-mediated dilation, SEM of Rc—Standard error of the men of regression coefficient. Multivariable logistic regression model for the probability of “FMD < mean value” in the study cohort. The table presents regression coefficients (Rc), standard errors of the regression coefficient (SEM of Rc), *p*-values, odds ratios (OR) for a one-unit increase in the predictor, and corresponding 95% confidence intervals.

**Table 6:** Results of estimation for the final model for probability of “carotid plaque score > mean value” obtained in logistic regression analysis.

		Model for: Probability of “Carotid Plaque Score > Mean Value”				
		Intercept	Physical Activity *	BMI (kg/m^2^)	Total Cholesterol (mg/dL)	Smoking **
Regression coefficient		−2.11	−2.39	4.42	0.41	4.01
SEM of Rc		0.79	1.08	2.01	0.09	1.58
*p*-value		0.01	0.04	0.04	0.01	0.02
Odds ratio (for unit change)		0.02	0.16	1.23	1.05	4.61
Confidence interval						
	+95%	0.01	0.11	1.31	1.03	3.97
	−95%	0.03	0.19	1.11	1.08	5.40

* ordinal variable, where: 0—low or high activity, 1—moderate activity, ** ordinal variable, where: 0—no, 1—yes, FMD—flow-mediated dilation, SEM of Rc—Standard error of the men of regression coefficient. Multivariable logistic regression model for the probability of “carotid plaque score > mean value” in the study cohort. The table presents regression coefficients (Rc), standard errors of the regression coefficient (SEM of Rc), *p*-values, odds ratios (OR) for a one-unit increase in the predictor, and corresponding 95% confidence intervals.

## Data Availability

The data are available from the authors upon reasonable request.
